# Evaluation of self-perception of awake bruxism in dentistry students – clinical case series

**DOI:** 10.1080/07853890.2021.1897344

**Published:** 2021-09-28

**Authors:** R. Ramos, R. Silva, P. Cebola, S. Félix, A. Mariz de Almeida

**Affiliations:** a Instituto Universitário Egas Moniz (IUEM), Egas Moniz Cooperativa de Ensino Superior, Caparica, Portugal; b Centro de Investigação Interdisciplinar Egas Moniz (CiiEM), Egas Moniz Cooperativa de Ensino Superior, Caparica, Portugal

## Abstract

**Introduction and Objectives:**

Bruxism is a repetitive muscular activity of the masticatory muscles where there may, or may not, exist dental contact and, depending on the adaptability of the individuals may cause lesions in the stomatognathic apparatus [1]. It is now possible to use the Ecological Momentary Assessment (EMA), which involves carrying out a self-report questionnaire about Awake Bruxism (AB) at various times of the day, in a random order in the subject's environment through smartphones [2]. The main objective of this study was to analyse auto perception of AB through the use of EMA in dentistry students.

**Materials and Methods:**

Ten (*n* = 10) dentistry students of the Instituto Universitário Egas Moniz, in Almada, Portugal, with access to a smartphone, were invited to participate in this study. All the assumptions of the Helsinki Declaration have been fulfilled and an informed consent for clinical case of Clinica Dentária Egas Moniz approved by the ethic commission of Instituto Universitário Egas Moniz. Initially, it was requested the signature of the informed consent. Then, each student answered two questionnaires (T_0_): The first was a specific bruxism questionnaire and the second was the “General Anxiety Disorder-7” (GAD7) questionnaire [3]. Finally, after a slideshow presentation about the BruxApp®, each student downloaded this app to their smartphone. The students were asked to use the app for 7 days, answering to, at least, 12 random alerts that were sent randomly during the day. At the end of 7 days-period the students were asked to answer again to the same 2 questionnaires (T_1_).

**Results:**

Unfortunately, we were not able to collect results from smartphones with the Android System® resulting in 5 dropouts. The results through the BruxApp® were: Relaxed: 74.1%; Teeth Contact: 15.52%; Teeth Clenching: 2.68%; Teeth grinding: 0%; Mandible Bracing: 7.62%. In what concerns the answers to the questionnaires: There was no major difference in GAD-7 questionnaire in T_0_ and T_1_ among students. Nevertheless, on the bruxism assessment questionnaires, in T_0_, four (*n* = 4) students did not have AB self-perception. However, in T_1_ all the students (*n* = 5) referred an increased self-perception of AB after using BruxApp® for 7 days. In total, the prevalence of AB among students was 24.8%.

**Discussion and conclusions:**

The total prevalence of AB (24.8%), was very similar to the study of Bracci [2] Also, teeth contact was shown to be the most frequent habit in both studies [2]. We can conclude, that in our study, the smartphone application BruxApp® allowed patients to have more information about AB, likewise increasing self-perception and contributing to establish a clearer idea of bruxism as an epidemiology. Moreover, it is important to refer that, besides being a pilot study, the loss of half of the participants due to failures with the Android System®, this is a severe limitation of this study.
